# Why results from Bayesian statistical analyses of clinical trials with a strong prior and small sample sizes may be misleading The case of the NICHD Neonatal Research Network Late Hypothermia Trial

**DOI:** 10.1111/apa.14800

**Published:** 2019-04-11

**Authors:** Lars Walløe, Nils Lid Hjort, Marianne Thoresen

**Affiliations:** ^1^ Division of Physiology Institute of Basic Medical Sciences University of Oslo Oslo Norway; ^2^ Division of Statistics and Biostatistics Department of Mathematics University of Oslo Oslo Norway; ^3^ Neonatal Neuroscience, Translational Health Sciences University of Bristol Bristol UK

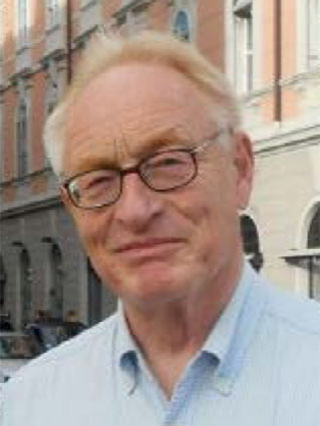

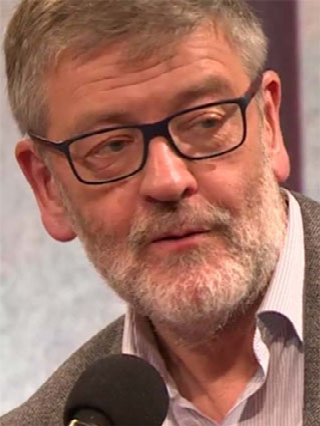

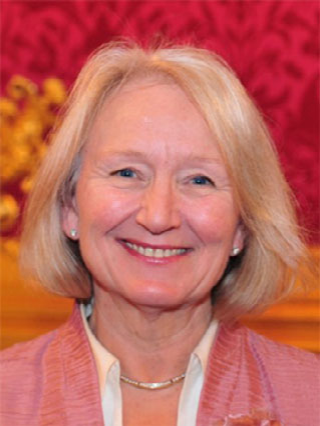

We would like to thank Laptook et al. 1 for their response to our ‘Major concerns about late hypothermia study’ 2. However, their response suggests that the difference between their opinion and ours arises because we are using frequentist statistics and they are using Bayesian. This is not the case. There is indeed general concern at present about the potential misuse of p‐values in frequentist statistical practice. We agree that generally in situations where a limited number of observations are available, the usual frequentist requirement for the significance probability to be lower than 0.05 is too strict. Results with a significance probability of 0.10 or even 0.15 may also give valuable information, and correspondingly a confidence interval (frequentist) or credibility interval (Bayesian) of 0.95 is sometimes too strict.

The heart of the matter is whether the observation that 19 of 78 neonates in group 1 (with cooling initiated in the time window from 6 to 24 hours after birth) showed adverse outcomes can be said to indicate that the associated probability p1 is smaller than the corresponding probability p0 in the control group, where 22 of 79 showed adverse outcomes. The original JAMA paper 3 discussed this in terms of the relative risk rr = p1/p0, and the question is whether there are any grounds to claim, with any meaningful confidence or credibility (to use the relevant frequentist and Bayesian terms), that rr is smaller than 1.

Our primary analysis was indeed frequentist, demonstrating that with sample sizes 79 and 78 there can be no meaningful statistical difference between the probability estimates 19/78 = 0.244 and 22/79 = 0.278. The close proximity of these two estimates can be assessed in several ways, including a p‐value far above the customary levels for significance (p = 0.75), and a confidence curve with the value rr = 1 in the middle with a 95 per cent confidence interval (0.51, 1.48) (see Fig. 1 in our previous communication (2)).

**Figure 1:**
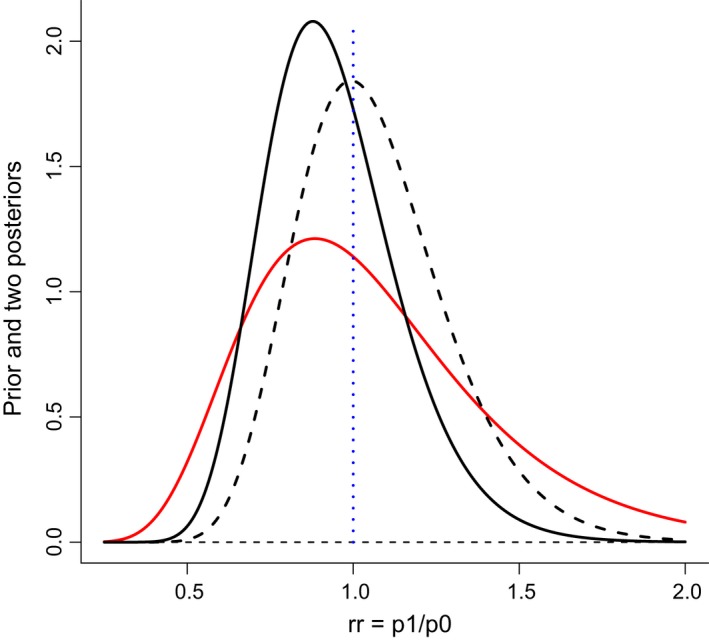
*Red curve:* The neutral prior probability distribution from Laptook et al. 3 with median rr = 1.0. The distribution is normal in log space with SD = 0.35. *Solid black curve:* Posterior distribution calculated from the prior and the results from the trial. *Dashed black curve:* Posterior distribution calculated from the hypothetical results were two infants with adverse outcome were moved from the non‐cooled group to the cooled group as a sensitivity test.

We have nothing against Bayesian analyses in general, and we agree that Laptook et al.'s ‘neutral’ unimodal prior with rr = 1 is sensible if no prior knowledge on late cooling is available. The detailed shape and especially the tails of this prior probability distribution are of course rather uncertain. As our figure clearly shows, Laptook et al.'s results give little support for the claim that p1 is smaller than p0 (i.e. that the relative risk parameter rr = p1/p0 above is smaller than 1). We have also performed a sensitivity analysis on the results of the trial. We moved two infants from the control group to the cooled group, so that the outcome was death or disability for 21 of 78 infants in the cooled group and 20 of 79 in the control group. These results would indicate a slightly better outcome for the control group, which is certainly possible if there is no real difference between the two groups.

The figure displays Laptook et al.'s Bayesian prior probability distribution (in red) and two posterior probability distributions (in black) for the rr parameter. The solid black curve is the posterior using the observed data, while the dashed black curve is the posterior using the hypothetical outcomes from the sensitivity analysis. The 95 per cent credibility intervals for rr are (0.61, 1.40) and (0.68, 1.58), respectively, for the two posterior distributions. rr = 1.00 is close to the middle (median) in all three cases. Even intervals with 70% credibility or less would cover rr = 1.00 in all three distributions, indicating that there is no real difference between the two groups.

In the JAMA paper Laptook et al. 3 write ‘The probability that death or disability in cooled infants was at least 1, 2, or 3% less than non‐cooled infants was 71, 64, and 56%, respectively’, and further: cooling ‘compared with non‐cooling resulted in a 76% probability of any reduction in death or disability’. Unfortunately, we have not been able to reproduce these numbers using Laptook et al.'s ‘neutral’ prior. According to our Bayesian calculations, the probability that death or disability in cooled infants was at least 1, 2, or 3% less than in non‐cooled infants was 64, 62 and 60%, respectively, and cooling resulted in a 65% probability of any reduction in death or disability. Using the posterior distribution from the sensitivity analysis, the probabilities were 41, 39, 37 and 43%, respectively. Two observations can be made from these numbers. There is always a positive probability that death and disability in cooled infants is less than in non‐cooled infants, even when the trial results indicate the opposite, but these probabilities vary greatly with small changes in the real outcome. We also see that the two posterior distributions are not very different from the prior distribution. In fact, they are heavily influenced by the prior and only to a limited degree by the results from the trial. The probabilities in the tails of the posterior distribution are also strongly influenced by the shape and width of the prior distribution, of which we have limited knowledge. The very uncertain tail probabilities in the posterior distribution should therefore definitely not be used as arguments for late cooling.

If the times when the cooling started had been given for all infants, other relevant statistical analyses could have been performed [e.g. logistic regression with time as a covariate or a Bayesian analysis with the results from late cooling of rat pups and foetal sheep as basis for a prior 2]. Although only tested in rats, one study showed that very severe injury increased more if cooling started 12 hour after the experimental insult 4. The current patient cohorts undergoing therapeutic hypothermia, including that in Laptook et al.'s 3 ‘Late hypothermia trial’ are all milder than the original trial cohorts that showed that therapeutic hypothermia was effective. As an example; in the 2005 NICHD whole body cooling trial, the non‐cooled group had 37% mortality and 40% disability in survivors 5. In the late hypothermia trial, the mortality in the non‐cooled group was 11, and 19% of the survivors had disabilities 3. We do not agree that there is evidence for suggesting starting hypothermia treatment late. Also, one does not know how high the risk for harm would be 6 if one expose rather mild HIE infants to 3 days of cooling and intensive care.

## Conflict of interest

The authors have no conflict of interests.

## Funding

Funding supporting work on therapeutic hypothermia was received by The Wellcome Trust, UK (RJ 3445 [059061/Z/ 99/A]), The Norwegian Research Council and Medical Research Council, UK (G0100126 (2002), G0801320 (2009)).

## References

[1] Laptook A , Tyson JE , Pedroza C , Shankaran S , Bell E , Goldberg R , et al. Response to A Different View concerning the NICHD Neonatal Research Network Late Hypothermia Trial. Acta Paediatr 2019; 108: 772–3. 30664824 10.1111/apa.14725 PMC6988380

[2] Walløe L , Hjort NL , Thoresen M . Major concerns about late hypothermia study. Acta Paediatr 2019; 108: 588–9. 30417430 10.1111/apa.14640 PMC6587492

[3] Laptook A , Shankaran S , Tyson JE , Munoz B , Bell E , Goldberg R , et al. Effect of therapeutic hypothermia initiated after 6 hours of age on death and disability among newborns with hypoxic‐ischemic encephalopathy: a randomised clinical trial. JAMA 2017; 318: 1550–60. 29067428 10.1001/jama.2017.14972 PMC5783566

[4] Sabir H , Scull‐Brown E , Liu X , Thoresen M . Immediate hypothermia is not neuroprotective after severe hypoxia‐ischemia and is deleterious when delayed by 12 hours in neonatal rats. Stroke 2012; 43: 3364–70. 22996953 10.1161/STROKEAHA.112.674481

[5] Shankaran S , Laptook AR , Ehrenkranz RA , Tyson JE , Scott A , McDonald BS , et al. Whole‐body hypothermia for neonates with hypoxic‐ischemic encephalopathy. N Engl J Med 2005; 353: 1574–84. 16221780 10.1056/NEJMcps050929

[6] El‐Dib M , Inder TE , Chalak LF , Massaro AN , Thoresen M , Gunn AJ . Should therapeutic hypothermia be offered to babies with mild neonatal encephalopathy in the first 6 h after birth? Pediatr Res 2019; 85: 442–8. 30733613 10.1038/s41390-019-0291-1

